# The Prokaryotic Community of Hypersaline Soils from the Odiel Saltmarshes: Culturomics Versus Metagenomics

**DOI:** 10.3390/life16081246

**Published:** 2026-07-27

**Authors:** Cristina Galisteo, Dáša Straková, Alicia García-Roldán, Rafael R. de la Haba, Cristina Sánchez-Porro, Antonio Ventosa

**Affiliations:** Department of Microbiology and Parasitology, Faculty of Pharmacy, University of Sevilla, 41012 Sevilla, Spain; crigalgomez@us.es (C.G.); dstrakova@us.es (D.S.); agroldan@us.es (A.G.-R.); rrh@us.es (R.R.d.l.H.)

**Keywords:** hypersaline soils, culturomics, metagenomics, halophilic microorganisms, microbial diversity

## Abstract

Hypersaline soils are poly-extreme terrestrial habitats characterized by high salinity, in some cases heavy-metal contamination, temperature fluctuations, and nutrient limitation. These conditions impose strong selective pressures, and many prokaryotic inhabitants still remain uncultured. Here, we conducted an extensive culturomics study of 549 isolates from the hypersaline soils of the Odiel Saltmarshes Natural Area (Southwest Spain) and compared the results with previously generated shotgun metagenomic datasets from the same environment in order to evaluate taxonomic composition, functional potential, and ecological representativeness. Cultivation across media containing 7.5%, 15%, and 25% (*w*/*v*) total salts yielded microorganisms belonging to three major phyla: *Pseudomonadota*, *Bacillota* (*Bacteria*) and *Halobacteriota* (*Archaea*). At the genus level, bacterial isolates were dominated by *Marinobacter*, *Halomonas*, and *Aquibacillus* at 7.5% (*w*/*v*) salinity, whereas extremely halophilic archaea, including *Halorubrum*, *Halogeometricum*, and *Haloarcula*, were predominantly recovered from media containing 25% (*w*/*v*) salts. Among the isolates, 57 strains displayed identity values < 98.65% for 16S rRNA gene sequence comparison, suggesting their putative status as new taxa. Comparison with metagenomic datasets showed that culture-dependent approaches successfully recovered the dominant haloarchaeal groups but missed some abundant bacterial phyla, such as *Gemmatimonadota*. Conversely, culturomics enabled the isolation of unknown species from the rare biosphere, including representatives of the novel genus *Terrihalobacillus*, which are typically detected at low abundance in metagenomic datasets. Together, these results demonstrate the complementarity of culturomics and metagenomics and provide an insight into the microbial communities inhabiting the hypersaline soils of the Odiel Saltmarshes.

## 1. Introduction

Hypersaline soils and their microbial communities have attracted increasing attention in recent years due to the progressive desertification of agricultural lands driven by anthropogenic activities and climate change [1,2,3,4,5,6,7,8,9]. These environments are often characterized not only by high salinity but also by elevated concentrations of heavy metals and other toxic compounds, which pose significant risks to microorganisms, plants, animals, and humans [10,11,12,13,14,15,16,17]. Such contamination is frequently associated with agricultural intensification, including the extensive use of fertilizers and pesticides, as well as improper disposal of industrial waste from chemical industries [18,19,20,21,22]. The coexistence of multiple environmental stressors makes hypersaline soils poly-extreme habitats that exert strong selective pressures on microbial life. Consequently, these environments represent ideal models for studying microbial diversity and the mechanisms underlying adaptation to extreme conditions [23,24,25].

The Odiel Saltmarshes Natural Area, situated at the confluence of the Tinto and Odiel rivers in Huelva (Southwest Spain), constitutes an example of such hypersaline ecosystems. Previous studies conducted by our research group have investigated the microbial diversity of these soils using culture-independent approaches. Early analyses based on pyrosequencing (Roche 454 GS FLX) provided the first insights into the composition of prokaryotic communities inhabiting these environments [26,27,28]. Although these studies revealed a high diversity of halophilic microorganisms, this sequencing technology is now considered obsolete. Advances in next-generation sequencing (NGS) technologies have since enabled more comprehensive analyses, providing greater sequencing depth, improved resolution, and a more detailed characterization of complex microbial communities [29]. Recent studies by our research team in the Odiel Saltmarshes have shifted towards high-throughput sequencing technologies, such as Illumina-based metagenomics, to advance our understanding of microbial diversity [30,31].

Despite these advances, a significant proportion of microorganisms in these hypersaline soils remain uncharacterized, highlighting the need for further investigation to comprehensively profile the microbiota of these extreme environments [30,31]. Culture-independent techniques provide valuable information on community composition and metabolic potential but do not allow direct investigation of the physiology or biotechnological potential of individual microorganisms. Therefore, cultivation-based approaches remain essential for obtaining pure cultures, validating genomic predictions, and enabling the formal description of novel taxa [32].

The aim of this study was to isolate halophilic bacteria and haloarchaea from the hypersaline soils of the Odiel Saltmarshes using culturomics approaches and to characterize the culturable prokaryotic diversity of these environments. Furthermore, the results obtained were compared with previously generated metagenomic datasets in order to evaluate the extent to which culture-dependent and culture-independent methods capture the microbial diversity present in these hypersaline soils.

## 2. Materials and Methods

### 2.1. Sampling Areas and Physico-Chemical Analysis

Two extensive samplings of hypersaline soils from the Odiel Saltmarshes Natural Area, a wetland ecosystem located on the southwestern coast of Spain, were conducted in January 2019 (M1) and July 2020 (M2) for culturomics studies (Figure S1). Sampling areas were selected due to the lack of vegetation. Salt crusted surface layer was discarded and homogenized subsurface stratum (2−4 cm), mixed with sterile spatula, was collected in Whirl-Pak^®^ (Sigma-Aldrich, Burlington, MA, USA) bags and conserved at 4 °C until laboratory processing within the next 24 h. In total, 14 soil samples were collected, as described in Table 1. Additionally, samples obtained in July 2020 (M2) were also used for metagenomic analyses, together with samples obtained during a subsequent sampling in June 2021 (M3, dataset not considered in the present study), as reported by Galisteo et al. [31]. In both samplings (M1 and M2), Area 1 has remained constant, as this site had previously been investigated by our research group using Roche 454 pyrosequencing technology [26,27,28]. The remaining sampling areas had not been studied previously.

Soil temperature was measured in situ using a Checktemp 2 thermometer (Electron Microscopy Sciences, Hatfield, PA, USA). For pH and electrical conductivity measurements, soil samples were diluted 1:5 in distilled water, and values were determined using a CRISON BASIC 20 pH meter and a CRISON 35+ conductometer, respectively. For the physicochemical composition analysis, 100 g of soils were submitted to the Innoagral Laboratory (Brenes, Sevilla, Spain). The analyses were measured as follows: aluminum, calcium, phosphorus, potassium, sodium, sulfur, chloride, copper, iron, manganese, and zinc concentrations were measured by atomic absorption spectroscopy; arsenic, cadmium, and nickel by Inductively Coupled Plasma–Mass Spectrometry (ICP-MS) (ThermoFisher Scientific, Waltham, MA, USA); cobalt and lead concentrations by Inductively Coupled Plasma–Optical Emission Spectroscopy (ICP-OES) (ThermoFisher Scientific, Waltham, MA, USA); nitrate, nitrite, and sulfate concentrations by ultraviolet-visible spectroscopy; carbon concentration by infrared combustion system; nitrogen concentration by Kjeldahl method; and clay, sand, silt, and texture by Bouyoucos method. The statistical significance between samplings was tested by ANOVA and Tukey tests, with Holm adjustment, after assessing their normality and homoscedasticity with Shapiro–Wilk’s and Bartlett’s tests (*p*-value < 0.05), respectively. Non-normal distributed data were tested by Kruskal–Wallis followed by pairwise Wilcoxon tests. These analyses were carried out in base “stats” R package v4.5.1.

### 2.2. Isolation of Strains

To recover the widest possible diversity of prokaryotic microorganisms, two culture media were employed: commercial Reasoner’s 2A (R2A) medium (Difco™) and Supplemented Minimal Medium (SMM), with the following composition (g L^−1^): sodium pyruvate, 0.11; casein digest, 0.50. For solid media, agar was supplemented to a final concentration of 2% (*w*/*v*). Previous studies identified these media as suitable for the cultivation of halophilic microorganisms associated with the presence of pyruvate in the media composition [33]. Both media were supplemented with 7.5%, 15%, or 25% (*w*/*v*) total salts using a 30% (*w*/*v*) stock seawater solution, as described by Subov [34], with the following composition (g L^−1^): NaCl, 195; MgCl_2_·6H_2_O, 32.5; MgSO_4_·7H_2_O, 50.8; CaCl_2_, 0.83; KCl, 5.0; NaHCO_3_, 0.17; and NaBr, 0.58. The pH was adjusted to 7.5. For prokaryotic isolation, 1 g of each soil sample was suspended in 10 mL of sterile 0.9% (*w*/*v*) NaCl solution and vigorously mixed. Serial dilutions were prepared up to 10^−5^, and aliquots from each dilution were inoculated onto R2A and SMM agar plates containing the three different salt concentrations. Plates were incubated at room temperature for up to three months to allow the growth of slow-growing halophilic microorganisms under aerobic conditions. Anaerobic cultivation was not considered, as the collected soils derived from subsurface stratum where the proliferation of strictly anaerobic microorganisms is unlikely. For each media and salinity, distinct colonies based on morphology aspects were subsequently isolated and purified through successive subculturing in the same media of reference, at 37 °C temperature for hastening the growing periods. Pure cultures were preserved at −80 °C in cryotubes using a 1:1 mixture of culture and 40% (*v*/*v*) glycerol.

### 2.3. DNA Extraction, Amplification, and Sequencing

Genomic DNA from isolated strains was extracted following the method described by Marmur [35], with modifications for small sample volumes. Amplification of the 16S rRNA gene was performed using primers 16F27 (AGA GTT TGA TCM TGG CTC AG) and 16R1492 (TAC GGY TAC CTT GTT ACG ACT T) for bacteria [36] and primers ArchF (TTC CGG TTG ATC CTG CCG GA) and ArchR (GGT TAC CTT GTT ACG ACT T) for archaea [37] and T100 Thermal Cycler (Bio-Rad, Hercules, CA, USA). The product was purified using the MEGAquick-spin™ Plus Fragment DNA Purification Kit (iNtRON Biotechnology, Seongnam, Republic of Korea) according to the manufacturer’s instructions. Initial 16S rRNA gene sequencing employed the aforementioned primers 16F27 or ArchF. When longer sequence coverage was required, additional primers were used, including 16R1492, 16F530 (GTG CCA GCA GCC GCG G), and 16R353 (ACT GCT GCC TCC CGT A) for bacteria, and ArchR, 16FB36 (GGA CTA CCA GGG TAT CTA), and 16FD34 (GGT CTC GCT CGT TGC CTG) for archaea. PCR products were sequenced using the Sanger chain-termination method by Stab Vida (Caparica, Portugal).

Partial and almost complete 16S rRNA gene sequences were assembled using ChromasPro software v1.5 (Technelysium Pty Ltd., Brisbane, Australia) followed by the manual correction of error or ambiguous positions. Taxonomic identification was performed by comparing the assembled sequences with those available in the EzBioCloud database [38]. Best hit was considered for taxonomy assignation to genus level. Because a single sequencing primer was typically used (either the bacterial primer 16F27 or the archaeal primer ArchF), most sequences ranged from approximately 800 to 1000 bp, representing partial rather than complete (~1500 bp) 16S rRNA gene sequences. To verify phylogenetic relationships among strains, particularly those belonging to the same genus, sequences were further compared using the BLASTn algorithm of the Basic Local Alignment Search Tool (BLAST v2.2.28+) [39], available through the National Center for Biotechnology Information (NCBI). Sequences with 16S rRNA percentage identity lower than 98.65% were checked for chimera with UCHIME2, implemented in Usearch v11 software [40], and ChimeraSlayer (release 20110519) [41]. SILVA v138.2 SSU Ref [42] and ChimeraSlayer Gold Database [41] were used as references.

### 2.4. Metagenomic and Genomic Reference Data Processing

Metagenomic sequences from the studied hypersaline soil collected during sampling M2 (July 2020) were available under the following GenBank accession numbers: SRS20604429 (sample M2_1A), SRS20617295 (sample M2_1B), SRS20617297 (sample M2_1C), SRS20617301 (sample M2_2A), SRS20604431 (sample M2_2B), SRS20604430 (sample M2_2C), SRS20617303 (sample M2_3A), SRS20617302 (sample M2_3B) and SRS20604432 (sample M2_3C). As described in Galisteo et al. [31], sequences were filtered using PRINSEQ v0.20.3 [43] and integrated in the SqueezeMeta v1.6.0 pipeline [44] for assembly with MEGAHIT v1.2.9 [45] and taxonomic annotation against the GenBank nr database [46] by DIAMOND v2.0.14.152 [47].

The ecological distribution of the 10 novel species recovered from the hypersaline soils of the Odiel Saltmarshes as part of this study and previously described was analyzed by fragment recruitment analysis against the aforementioned nine metagenomes previously sequenced from the M2 sampling point [31]. We accessed the publicly available reference genomes for the species *Aquibacillus salsiterrae* 3ASR75˗54^T^ (GCA_028416595.1) [48], *Pseudidiomarina terrestris* 1APP75˗27a^T^ (GCA_034787225.1) [49], *Fodinibius salsisoli* 1BSP15˗2V2^T^ (GCA_026229185.1) [50], *Terrihalobacillus insolitus* 3ASR75˗11^T^ (GCA_028416575.1) [48], *Natrinema salsiterrestre* S1CR25˗10^T^ (GCA_029502005.1) [51], *Haloarcula terrestris* S1AR25˗5A^T^ (GCA_031624315.1) [52], *Haloarcula salsiterrae* S1CR25˗12^T^ (GCA_031624395.1), *Haloarcula onubensis* S3CR25˗11^T^ (GCA_031624605.1) [53,54], *Halogeometricum salsisoli* S1BR25˗6^T^ (GCA_031624495.1) and *Halogeometricum luteum* S3BR5˗2^T^ (GCA_031624635.1) [52]. The 16S rRNA gene sequences from the genomes were masked in order to avoid their highly conserved nature. Metagenomic reads ≥30 bp were BLASTn v2.2.28+ [39]-searched, independently, against each genome. BLASTn results were filtered as recommended by Mehrshad et al. [55] and normalized into RPKG (reads recruited per kilobase of genome per gigabase of metagenome), as proposed by Nayfach and Pollard [56].

## 3. Results and Discussion

### 3.1. Physicochemical Characteristics of the Hypersaline Soils

**Figure 1:**
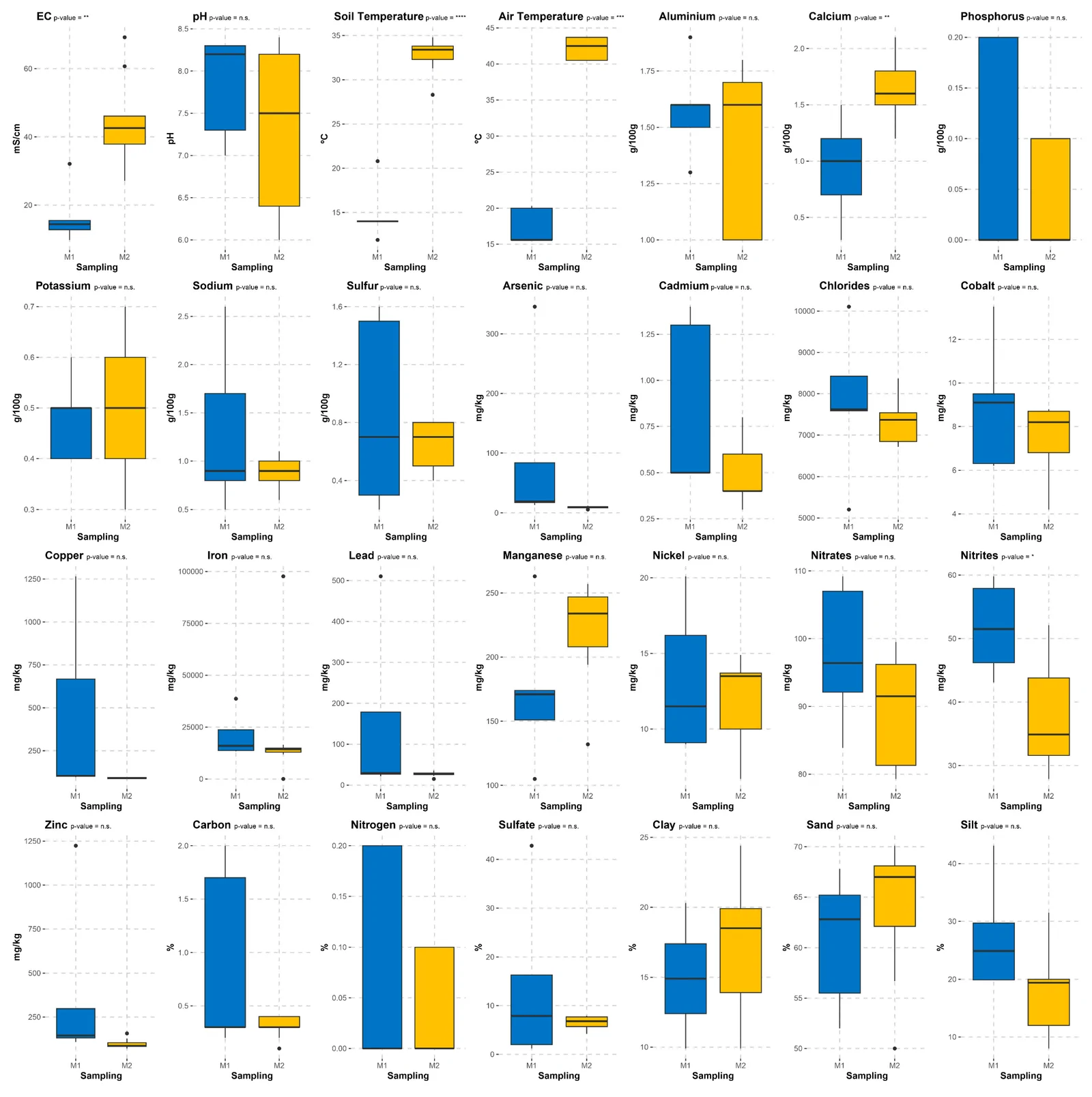
Measured values of physicochemical parameters of the sampled soils from Odiel Saltmarshes, by sampling. n.s.—*p*-value > 0.05; *—*p*-value ≤ 0.05; **—*p*-value ≤ 0.01; ***—*p*-value ≤ 0.001; ****—*p*-value ≤ 0.0001 (Wilcoxon tests or one-way ANOVA and Tukey tests, with Holm adjustment).

The delimitation of saline conditions in terrestrial environments is less standardized than in aquatic systems. In general, soils with an electrical conductivity above 4 mS cm^−1^ at 25 °C in a saturated soil extract are considered saline [57,58]. Based on this criterion, all soils analyzed in this study can be classified as saline to hypersaline, with EC_1:5_ values ranging from 9.81 to 69.15 mS cm^−1^ (Figure 1; Table S1). The lowest conductivity values were recorded in the M1 sampling (January 2019), when soil temperatures ranged from 11.9 to 20.8 °C. In contrast, samples collected during the M2 sampling (July 2020) showed markedly higher soil temperatures (32.1–37.2 °C) and, in parallel, higher conductivity values (27.06–69.15 mS·cm^−1^) (Figure 1; Table S1). This pattern is consistent with the strong seasonal influence on these soils, where increased evaporation during the warmer months likely concentrates salts in the surface layers. Similar conductivity values were previously reported for soils from the Odiel Saltmarshes, with EC values ranging from 5.96 to 61.02 mS cm^−1^ in samples collected in July 2015 [30], indicating that the high salinity of this system is a stable environmental feature.

Soil pH also varied substantially among sampling areas (Figure 1; Table S1). Area 1, sampled in both M1 and M2, showed consistently slightly alkaline values, ranging from pH 8.16 to 8.40, whereas samples M1_2A (Area 2 from sampling M1) and M1_3A (Area 3 from sampling M1) were closer to neutrality, with pH values of 7.25 and 6.99, respectively. In sampling M2, samples from Area 2 remained within a near-neutral range (pH 7.44–7.73), while Area 3 displayed the lowest pH values of all samples (pH 6.01–6.44), representing the only slightly acidic soils included in this study. Based on these results and the lack of statistical significance (Figure 1), culture media were prepared at an intermediate pH of 7.5 to accommodate the overall pH range observed in the sampled soils. Our previous analysis did not correlate changes in the prokaryotic community with variations in the soil physicochemical features among samplings and areas [31].

Previous studies have shown that the Odiel River and surrounding sediments have been strongly impacted by mining and industrial activity, resulting in elevated concentrations of arsenic, cadmium, copper, lead, and zinc [59,60]. According to reference values proposed by the Department of the Environment of the Autonomous Community of Andalucia for uncontaminated soils in natural areas, threshold ranges are 2–5 mg·kg^−1^ for arsenic, 0.4–0.8 mg·kg^−1^ for cadmium, 17–100 mg·kg^−1^ for copper, 10–50 mg·kg^−1^ for lead, and 10–70 mg·kg^−1^ for zinc [61]. Based on these criteria, all soil samples exceeded the reference range for arsenic and zinc. In addition, samples M1_2A and M1_3A also exceeded the limits for cadmium and lead, while samples M1_1B, M1_1C, M1_2A, and M1_3A exceeded the upper reference value for copper (Figure 1; Table S1). Thus, the microbial communities inhabiting these soils are exposed not only to high salt concentrations but also to elevated levels of heavy metals.

### 3.2. Recovery of Prokaryotic Diversity by Culturomics

Approximately 4000 isolates were obtained in pure culture from the two sampling campaigns. After incubation, media supplemented with the lowest salt concentration presented the highest culturable prokaryotic abundances (1–3 × 10^5^ CFU g^−1^, R2A 7.5%; 6 × 10^4^–1 × 10^5^ CFU g^−1^, SMM 7.5%), followed by intermediate salinity (7 × 10^3^–1 × 10^5^ CFU g^−1^, R2A 15%; 6 × 10^4^–1 × 10^5^ CFU g^−1^, SMM 15%) and lowest abundance for highest salt concentration (1–3 × 10^3^ CFU g^−1^, R2A 25%; 4–8 × 10^4^ CFU g^−1^, SMM 25%). Based on morphology aspects and aiming to obtain the greater, as well as putative unknown diversity, 549 isolates were selected for taxonomic identification based on partial 16S rRNA gene sequencing. Of these, 452 strains (82.3%) were assigned to the domain *Bacteria*, whereas 97 strains (17.7%) belonged to the domain *Archaea*. This distribution contrasts with metagenomic analyses of the same soils, which showed an approximately balanced representation of both domains [31]. The predominance of bacteria among cultured isolates therefore reflects a clear cultivation bias, likely associated with the broader physiological flexibility of many bacterial taxa in contrast to the more demanding nutritional or physicochemical requirements, as well as growing periods, of numerous haloarchaea [62].

**Figure 2:**
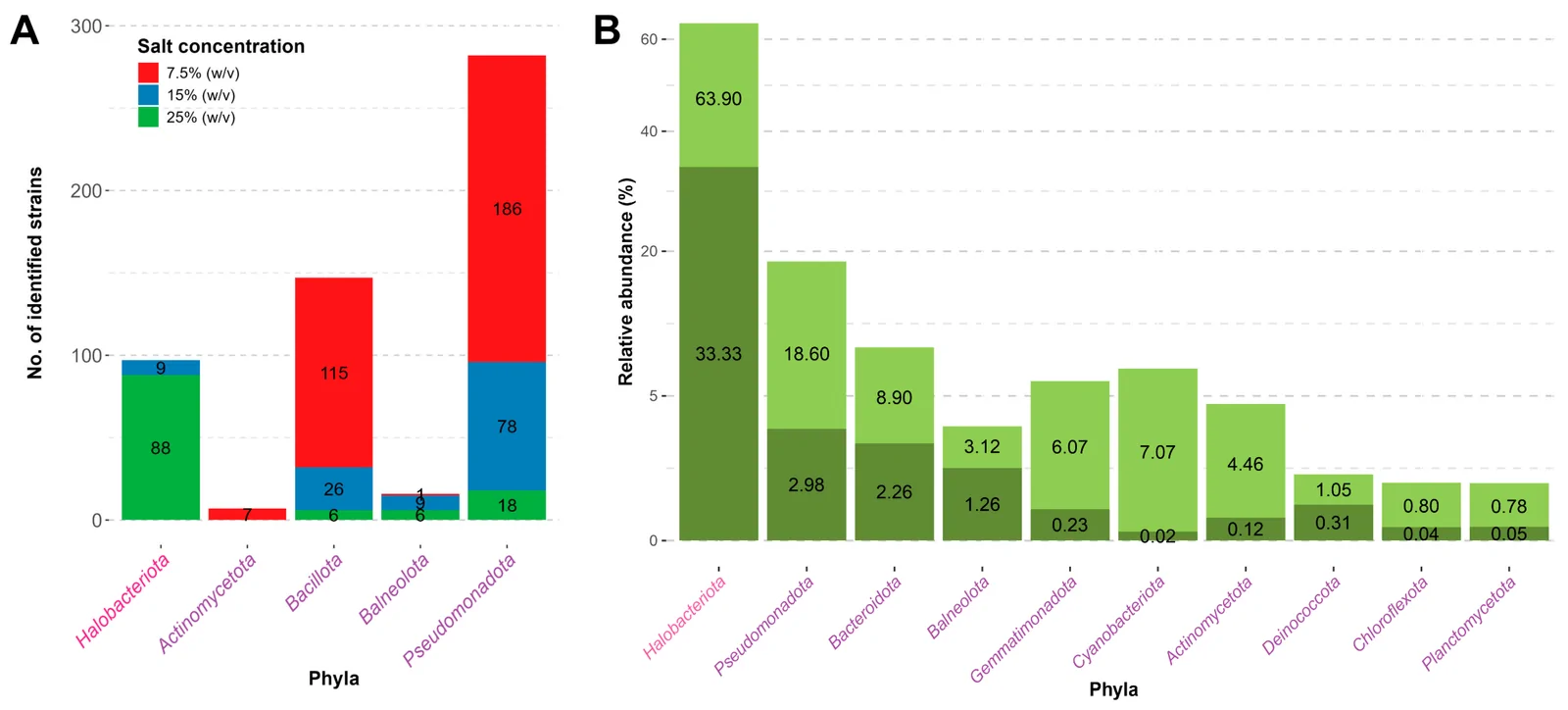
(**A**) Distribution of the 549 isolates from the hypersaline soils of the Odiel Saltmarshes (M1 and M2) across different phyla, sorted by salt concentration of the isolation media. (**B**) Maximum (light green) and minimum (dark green) relative abundance (%) of the main phyla detected in the nine *shotgun* metagenomic contigs from sampling M2, further analyzed in Galisteo et al. [31]. Pink-colored names belong to the archaeal phylum, whereas purple-colored names belong to the bacterial phyla.

At the phylum level, most bacterial isolates belonged to *Pseudomonadota* (282 strains) and *Bacillota* (147 strains), whereas the archaeal isolates (97 strains) were exclusively assigned to *Halobacteriota*. In addition, smaller numbers of isolates affiliated with *Balneolota* (16 strains) and *Actinomycetota* (7 strains) were recovered (Figure 2A). Most of these phyla were also detected in the metagenomic datasets from the same environment (Figure 2B) previously studied [31], and other hypersaline environments [4,8,63,64,65,66,67,68,69]. Moreover, strains from the phyla *Pseudomonadota*, *Bacillota*, and *Actinomycetota* have been previously isolated from hypersaline soils located at Rambla Salada (Murcia, Spain), Great Salt Plains (Oklahoma, USA), Heilongjiang province (China), and coastal areas of Korea [70,71,72,73]. In comparison with the metagenomic studies, *Bacillota* was strongly represented among cultured isolates (Figure 2A), suggesting that members of this phylum were favored by the cultivation media and incubation conditions used. Conversely, *Gemmatimonadota*, which was one of the most abundant phyla detected by metagenomics (Figure 2B), was not recovered by culturomics (Figure 2A). This phylum is fastidious to growth [74,75], and it is underrepresented in laboratory cultures despite the variety of ecological niches that it inhabits [76,77,78]. In this study, regular conditions were used for isolation in order to accommodate the greatest diversity of prokaryotes, which may explain the absence of representative *Gemmatimonadota* species among the isolates. Moreover, our result in the culturable community diversity is reshaped by the success in subculturing the isolates and/or 16S rRNA amplification with universal primers. Regardless, there is a range of diversity within these five main culturable phyla (*Pseudomonadota*, *Bacillota*, *Halobacteriota*, *Balneolota*, and *Actinomycetota*). The distribution of the isolates at lower taxonomic ranks is further detailed in Figure S2. Henceforth, our results and further discussion will focus on the taxonomy genus level.

**Figure 3:**
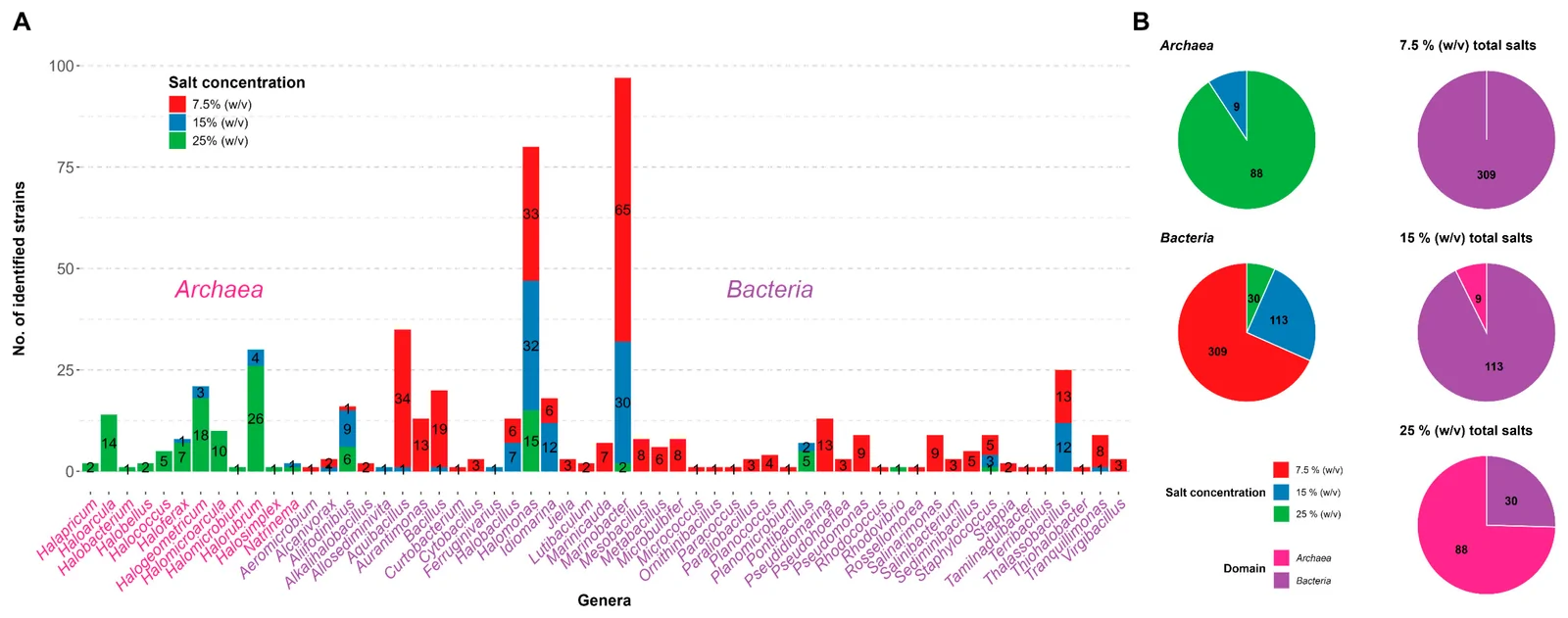
(**A**) Genus-level classification of the 549 isolates from the hypersaline soils of the Odiel Saltmarshes (M1 and M2), categorized according to the salt concentration of the cultivation media used for their isolation. Pink-colored names belong to genera from the domain *Archaea*, whereas purple-colored names belong to genera from the domain *Bacteria*. (**B**) Domain distribution of the isolates regarding the total salt concentration supplementation in their isolation media.

In our culturomics study at the genus level, the most frequent bacterial isolates belonged to *Marinobacter* (97 strains), *Halomonas* (80 strains), and *Aquibacillus* (35 strains) (Figure 3A). Although approximately 70% of the bacterial strains were isolated from media supplemented with 7.5% (*w*/*v*) total salts (Figure 3B), the genus *Halomonas* was found at all the studied salinities and comprised half of the bacterial strains isolated at 25% (*w*/*v*) total salts (Figure 3). In contrast, haloarchaeal strains, mainly affiliated with the genera *Halorubrum* (30 strains), *Halogeometricum* (21 strains), and *Haloarcula* (14 strains) (Figure 3A), were predominantly isolated on media supplemented with 25% (*w*/*v*) total salts as most of the archaeal isolates (Figure 3B). This salinity-dependent distribution is consistent with the known physiology of these groups, as dominant haloarchaeal genera are typically extreme halophiles whose biological mechanisms are adapted to higher ionic concentrations inside the cell [79], whereas many bacterial halophiles tolerate a broad range of salt concentrations. Particularly, many species of the genus *Halomonas* can grow at a wide NaCl concentration range [80]. These findings also agree with the metagenomic analysis of the same soils, which identified members of the archaeal families *Haloferacaceae* (harboring the genera *Halorubrum* and *Halogeometricum*), *Haloarculaceae* (comprising the genus *Haloarcula*), and *Halobacteriaceae* among the dominant taxa [31]. Together, these findings indicate that the culture-dependent strategy carried out in this study was effective in recovering the dominant haloarchaeal groups present in the hypersaline soils of the Odiel Saltmarshes.

### 3.3. Identification of Isolates Representing Potential Novel Taxa

**Figure 4:**
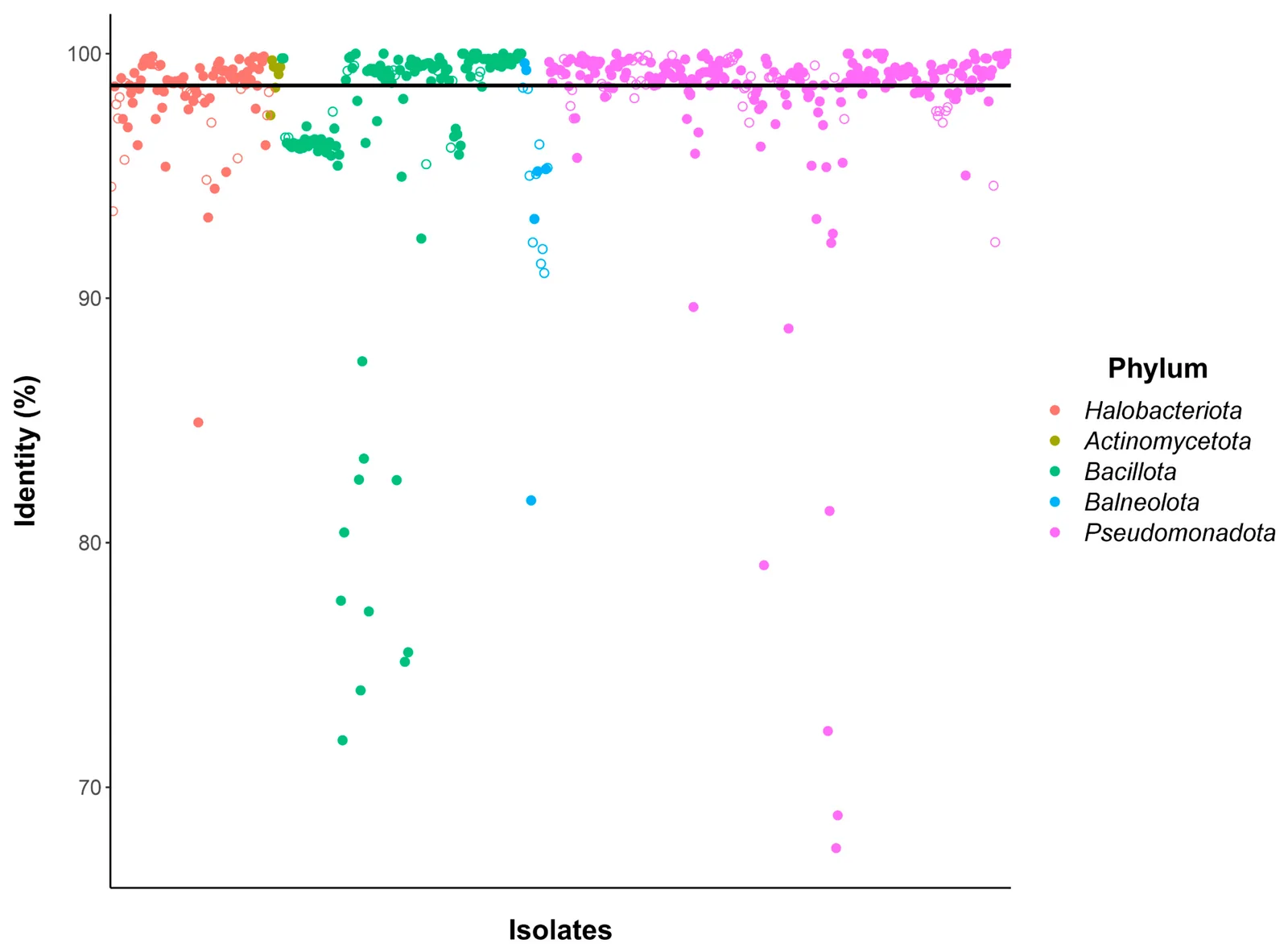
Best-hit identity percentages for the 16S rRNA gene sequences of the isolates from the hypersaline soils of the Odiel Saltmarshes (M1 and M2), sorted by phyla. Horizontal line delimits the 98.65% identity threshold for delineation of new species. Empty circles represent 16S rRNA gene sequences ≥ 1395 bp length.

The partial 16S rRNA gene sequence identity allowed the tentative affiliation of the isolates at the species level by using cutoff values ≥ 98.65% [81,82,83], widely accepted for delineation of prokaryotic species. For a more precise identification, almost complete 16S rRNA gene sequences were subsequently obtained for isolates whose partial 16S rRNA gene sequences showed identity values below the threshold [81,82,83] to recognized type strains in the EzBioCloud database. Still, many of the isolated strains showed an identity percentage below the aforementioned cutoff (Figure 4). It must be noted that some genera within the haloarchaeal phylum *Halobacteriota*, such as *Haloadaptatus*, *Haloarcula*, *Halobaculum*, *Halomicrobium*, *Halorubrum*, and *Halosimplex*, harbor multiple highly divergent copies of the 16S rRNA gene [84,85,86]. Consequently, the PCR amplification and sequencing generated a chimera of all these copies that could not be correctly assigned to the species level based on the identity values. None of the sequences with percentages below 98.65% were predicted as chimera.

Isolates with ≥1395 bp-long 16S rRNA gene sequences and identity values < 98.65% were considered candidates for further taxonomic characterization. A total of 57 strains met this criterion (Table S2). Among the haloarchaeal candidates, the strains were affiliated with the genera *Haloarcula* (six strains), *Halogeometricum* (four strains), *Halorubrum* (two strains), *Halomicroarcula* (three strains), *Natrinema* (two strains), *Halapricum* (two strains), *Haloferax* (one strain), and *Halobellus* (one strain). The bacterial candidates were assigned to the genera *Aliifodinibius* (10 strains), *Pseudidiomarina* (six strains), *Aquibacillus* (four strains), *Aurantimonas* (three strains), *Halomonas* (two strains), *Jiella* (two strains), *Lutibaculum* (two strains), *Marinicauda* (two strains), *Marinobacter* (one strain), *Ornithinibacillus* (one strain), *Pseudohoeflea* (one strain), *Tamilnaduibacter* (one strain), and *Thiohalobacter* (one strain).

Although not all 57 candidate strains could be fully characterized within the timeframe of this study, they were preserved for future characterization. Detailed comparative genomic, phenotypic, and chemotaxonomic analyses were completed for 10 representative strains, resulting in the formal description of several new taxa. Among *Bacteria*, the new proposals included *Aquibacillus salsiterrae* sp. nov. (isolate 3ASR75-54^T^) [48], *Pseudidiomarina terrestris* sp. nov. (isolate 1APP75-27a^T^) [49], *Fodinibius salsisoli* sp. nov. (isolate 1BSP15-2V2^T^), which led to the reclassification of species previously classified within the genus *Aliifodinibius* [50] and *Terrihalobacillus insolitus* gen. nov., sp. nov. (isolate 3ASR75-11^T^) [48].

Among *Archaea*, the study led to the description of the new species *Natrinema salsiterrestre* (isolate S1CR25-10^T^) [51], *Haloarcula terrestris* (isolate S1AR25-5A^T^) [52], *Halomicroarcula saliterrae* (isolate S1CR25-12^T^) (now reclassified as *Haloarcula saliterrae*), *Halomicroarcula onubensis* (isolate S3CR25˗11^T^) (now reclassified as *Haloarcula onubensis*) [53,54], *Halogeometricum salsisoli* (isolate S1BR25˗6^T^), and *Halogeometricum luteum* (isolate S3BR25˗2^T^) [87]. Our previous descriptive studies of these novel taxa [48,49,50,88] included genome-based analyses that revealed adaptive traits consistent with the poly-extreme nature of the sampled habitat, including mechanisms related to osmoadaptation through both “salt-in” and “salt-out” strategies, as well as genes and functions associated with heavy-metal tolerance. These results based on isolated strains and whole genome sequencing matched with the functional profile of the environmental microbiota inferred from shotgun metagenomic sequencing, which suggested a remarkable tolerance to arsenic for the haloarchaeal community [31], as confirmed by wet-lab experiments [88].

**Figure 5:**
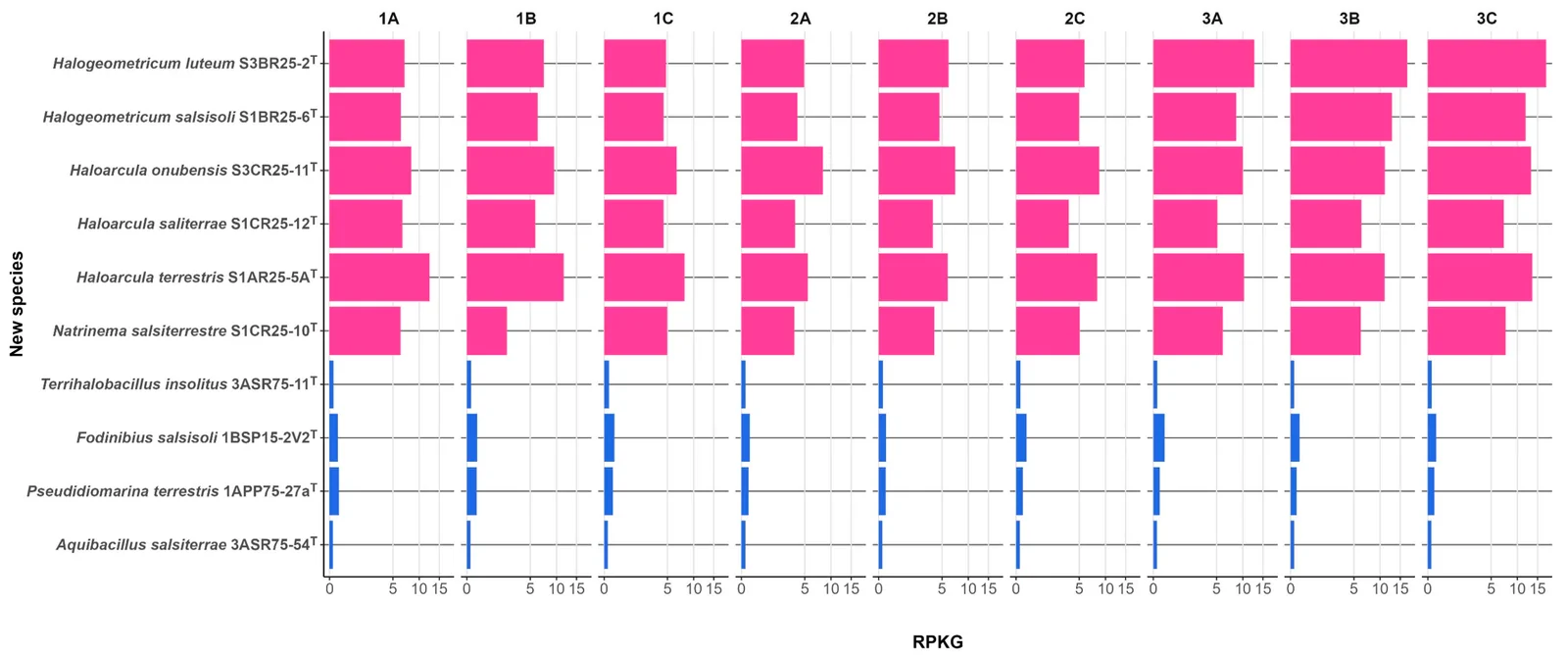
Relative abundance represented as RPKG of isolates from hypersaline soils of the Odiel Saltmarshes already described as novel species against the metagenome dataset M2 [21]. Square root transformation was performed for the *X*-axis in order to better visualize low values.

Regarding the distribution of novel species in the habitats, read-recruitment analysis showed a lower abundance of the bacterial new species in comparison to the haloarchaeal new species (Figure 5). Furthermore, in-depth analysis had established some of these bacteria as members of a rare biosphere [48]. Based on the metagenomic sequencing, these species could not be reconstructed or even identified at read/contig level, whereas traditional isolation recovered multiple strains of these previously uncovered species, such as *Aquibacillus salsiterrae*, *Terrihalobacillus insolitus*, and *Pseudidiomarina terrestris* (Figure 5) [48,49]. Overall, the differences observed between culturomics and metagenomics support the view that both approaches are complementary and together provide a more complete picture of the prokaryotic diversity inhabiting the hypersaline soils of the Odiel Saltmarshes.

On the other hand, both techniques unveiled the diversity of species within the phylum *Balneolota* in the hypersaline soils of the Odiel Saltmarshes. The metagenomic sequence analysis included this phylum among the most abundant in this ecosystem (Figure 2B) [31], and its child taxon *Balneolaceae* was the most represented family within the high- and medium-quality Metagenome-Assembled Genomes (MAGs) in this environment [89]. Moreover, the species “*Candidatus* Halalkalibaculum distributum” was proposed based on the phylogenomic analysis and functional traits inferred from its high-quality reconstructed MAG [89]. Although only this novel *Candidatus* species within the *Balneolaceae* was proposed due to the medium-to-low-quality of the other MAGs, the phylogenomic analysis of the total of 51 MAGs assigned to the family suggested multiple undescribed species and, even, novel genera [89]. In this study, 16 isolates affiliated to the phylum *Balneolota* were recovered from the three studied salt concentration-supplemented media (Figure 2A). Ten of them could not be assigned to any known *Balneolota* species based on their 16S rRNA gene sequence. Furthermore, their best-hit identity ranged from 91.03 to 98.61% (Table S2) for species within the genus *Aliifodinibius* (currently, *Fodinibius*). Isolate 1BSP15-2V2 was successfully subcultured, which favored its description as the new species *Fodinibius salsiterrae* [50]. The other isolates showed poor growth for the same culture conditions after isolation. Species from the family *Balneolaceae* seem to present a relatively complex genome (sometimes over 5 Mb size) with high-costing metabolic pathways such as biotin biosynthesis [50,90]. Thus, the intricacy of its metabolisms may not be rightly covered by media composition.

## 4. Conclusions

The hypersaline soils of the Odiel Saltmarshes represent a poly-extreme environment characterized by high salinity, physicochemical heterogeneity, and significant heavy-metal contamination. These conditions create strong selective pressures that shape the structure and diversity of the microbial communities inhabiting these soils. The culturomics approach employed in this study enabled the recovery of a large collection of prokaryotic isolates, revealing a diverse assemblage of halophilic bacteria and haloarchaea. Most isolates belonged to the bacterial phyla *Pseudomonadota* and *Bacillota* and the archaeal phylum *Halobacteriota*, with clear differences in salinity preferences between bacterial and archaeal taxa. Comparison with previously obtained metagenomic datasets from the same hypersaline habitat demonstrated that culture-dependent and culture-independent approaches capture complementary aspects of microbial diversity. While metagenomics revealed the dominant taxonomic groups present in these soils, culturomics enabled the isolation of both abundant taxa and members of the microbial rare biosphere that remain underrepresented in sequencing-based surveys. Notably, several isolates displaying low 16S rRNA gene sequence identity to previously described taxa were identified. Subsequent detailed analyses of selected strains enabled the characterization and description of several novel bacterial and archaeal species. Genomic analyses of these taxa revealed adaptive traits consistent with the poly-extreme nature of the Odiel Saltmarshes, including mechanisms related to osmoadaptation and resistance to heavy metals. Interestingly, read-recruitment analysis showed that the new bacterial species were much less abundant in the hypersaline soils studied, in contrast to their halobacterial counterparts. Overall, this study demonstrates that integrating culturomics with metagenomics provides a more comprehensive understanding of microbial diversity in environments with complex diversity, particularly hypersaline soils.

## Figures and Tables

**Table 1:** Sampling locations and coordinates of hypersaline soil samples collected in the Odiel Saltmarshes. Letters A, B, and C refer to biological replicates in the same sampling area.

Sampling	Date	Area	Sample	Coordinates
M1	January 2019	1	M1_1A	37°12′25″ N 6°57′54″ W
			M1_1B	
			M1_1C	
		2	M1_2A	37°12′09″ N 6°57′00″ W
		3	M1_3A	37°17′07″ N 6°57′46″ W
M2	July 2020	1	M2_1A	37°12′25″ N 6°57′54″ W
			M2_1B	
			M2_1C	
		2	M2_2A	37°12′30″ N 6°57′33″ W
			M2_2B	
			M2_2C	
		3	M2_3A	37°13′17″ N 6°57′44″ W
			M2_3B	
			M2_3C	

## Data Availability

The partial and or complete 16S rRNA gene sequences are available under the accession numbers OQ569934–OQ570418 (GenBank repository).

## Supplementary Materials

The following supporting information can be downloaded at https://www.mdpi.com/article/10.3390/life16081246/s1, Figure S1: Location of sampling areas. Figure S2: Taxonomic classification of the 549 isolates within the phylum, class, order, family and genus rank. Arrows width is proportional to the number of isolates for each taxonomic group; Table S1: Physicochemical parameters of hypersaline soils from Odiel Saltmarshes analyzed in this study; Table S2: Isolates with almost complete 16S rRNA gene sequences with identity percentages below the 98.65% established for species delineation against the type strains of prokaryotic species from EzBioCloud database.
